# Interleukin‐6 and thrombopoietin concentrations in dogs with carcinoma with and without thrombocytosis

**DOI:** 10.1111/jvim.16317

**Published:** 2021-12-08

**Authors:** Adrienne Cheney, Andrew D. Woolcock, Abhijit Mukhopadhyay, Deborah Knapp, George E. Moore

**Affiliations:** ^1^ Department of Veterinary Clinical Sciences, College of Veterinary Medicine Purdue University West Lafayette Indiana USA; ^2^ Department of Veterinary Administration, College of Veterinary Medicine Purdue University West Lafayette Indiana USA

**Keywords:** biomarkers, carcinoma, thrombocytosis

## Abstract

**Background:**

Carcinoma‐associated thrombocytosis involves tumor production of mediators such as interleukin‐6 (IL‐6) and thrombopoietin (TPO) that increase thrombopoiesis and may play a role in tumor evasion and metastasis. Carcinoma‐associated thrombocytosis is described in people, but has not been described in dogs.

**Hypothesis/Objectives:**

Evaluate the concentrations of IL‐6 and TPO in dogs diagnosed with carcinoma with or without thrombocytosis. We hypothesized that IL‐6 and TPO concentrations would be higher in dogs with carcinoma compared to healthy dogs, and that IL‐6 and TPO concentrations would be higher in dogs with carcinoma and thrombocytosis when compared to dogs with carcinoma and normal platelet counts.

**Animals:**

One‐hundred sixteen dogs: 63 with carcinoma and 53 healthy control dogs.

**Methods:**

Complete blood count was performed in all dogs, and they were stratified for sub‐group analysis based on the presence or absence of thrombocytosis (platelet count > 500 103/µL). Serum TPO and IL‐6 concentrations were measured by ELISA. Results of selected numeric variables were compared using Wilcoxon rank sum tests for pairwise comparisons. A value of *P* < .05 was considered significant.

**Results:**

Twelve of the dogs with carcinoma (12/63, 19.0%) and none of the healthy control dogs (0%) had thrombocytosis. Thrombopoietin concentrations (median [range]) were significantly higher in dogs with carcinoma when compared to controls (87.42 pg/mL [0 to >600] vs 15.99 pg/mL [0 to >600], *P* < .001). Interleukin‐6 concentrations (median [range]) were not different between dogs with carcinoma and healthy control dogs (9.70 pg/mL [0‐181.53] vs 3.03 pg/mL [0‐280.77], *P* = .15). In dogs with carcinoma, the TPO and IL‐6 concentrations were not different between dogs with thrombocytosis and dogs with normal platelet count.

**Conclusions and Clinical Importance:**

Thrombopoietin concentrations were significantly higher in dogs with carcinoma, regardless of platelet count. Thrombopoietin is likely to be 1 of multiple factors that can impact platelet number, production, and consumption in dogs with carcinoma.

**Abbreviations**
CKD: chronic kidney disease
DM: diabetes mellitus
IL‐6: interleukin‐6
MPV: mean platelet volume
TNF‐α: tumor necrosis factor α
TPO: thrombopoietin

## 1 INTRODUCTION

In health, platelets respond to injury, and provide hemostasis as well as a scaffolding for angiogenesis and vessel repair.^1^, ^2^ Platelets also have a role in other important pathways such as modulating the immune response, microbial resistance, and cellular regeneration.^1^, ^2^ Consumption of platelets leading to thrombocytopenia is described in a number of inflammatory and neoplastic disease processes.^3^ Additionally, thrombocytosis has been described in a number of disease states, including endocrine diseases, heart disease, infectious disease, and cancers.^3^, ^4^, ^5^, ^6^ Expanded knowledge of platelets and their interactions in disease states has led to investigation into maladaptation of platelet synthesis and function. One maladaptation is paraneoplastic thrombocytosis, in which tumor production of inflammatory mediators or stimulatory molecules leads to an increase in platelet count, which may aid in metastasis and tumor evasion of the immune system.^2^, ^6^, ^7^ Several different tumor types in people have been associated with paraneoplastic thrombocytosis, many of which are carcinomas resulting in the term carcinoma‐associated thrombocytosis.^5^, ^6^, ^7^, ^8^, ^9^, ^10^, ^11^, ^12^, ^13^, ^14^, ^15^, ^16^ Carcinoma‐associated thrombocytosis is rarely described in dogs.^4^, ^17^, ^18^

Ultrastructure studies in people have shown tumor emboli distant from primary tumors surrounded by platelets, suggesting that tumors have the ability to activate and aggregate platelets.^6^ Further evaluation of platelet interaction with tumor cells has shown that platelets can form a cloak around tumor cells to protect them from the immune system.^6^, ^7^, ^19^ Additionally, activated platelets stimulate angiogenesis, bringing additional blood flow to tumors. Malignancies are thought to interact with platelets through numerous mechanisms including tumor expression of platelet receptors and tumor secretion of thrombin and tumor‐specific cytokines, specifically interleukin‐6 (IL‐6).^6^, ^7^, ^20^, ^21^

In carcinoma‐associated thrombocytosis, it is hypothesized that tumor production of IL‐6 directly leads to the generation of hepatic thrombopoietin (TPO), which stimulates megakaryocytic generation of platelets.^20^, ^22^, ^23^, ^24^, ^25^, ^26^, ^27^, ^28^, ^29^ Interleukin‐6 concentrations are increased in people with ovarian, pulmonary, and urothelial carcinoma.^5^, ^6^, ^7^, ^8^, ^9^, ^10^, ^11^, ^12^, ^13^, ^14^, ^15^, ^16^, ^20^, ^21^ Carcinoma‐associated thrombocytosis has been associated with and predictive of neoplastic progression in human patients with ovarian cancer, and is an independent prognostic factor in endometrial, pancreatic, esophageal, and pulmonary carcinoma.^5^, ^6^, ^7^, ^8^, ^9^, ^10^, ^11^, ^12^, ^13^, ^14^, ^15^, ^16^, ^20^, ^21^

Thrombocytosis has been evaluated retrospectively in dogs and cats, and neoplasia has been identified as a primary or concurrent diagnosis in 17% to 55% of these patients.^3^, ^4^, ^17^, ^18^ In dogs with cancer and thrombocytosis, carcinoma was the most common tumor type diagnosed. Carcinoma‐associated thrombocytosis has not been investigated in dogs. Carcinoma‐associated thrombocytosis represents a novel mechanism for tumor evasion in dogs. There is continued interest in exploring inflammatory mediators or tumor biomarkers that could improve prognostication.

Our aim was to evaluate concentrations of IL‐6 and TPO in dogs with carcinoma with respect to their platelet count, and to compare the findings to those in normal, healthy dogs. Our hypothesis was that concentrations of IL‐6 and TPO would be significantly higher in dogs with carcinoma when compared to healthy dogs. We further hypothesized that dogs with carcinoma and concurrent thrombocytosis would have higher concentrations of IL‐6 and TPO when compared to dogs with carcinoma and normal platelet counts.

## 2 MATERIALS AND METHODS

### 2.1 Animals

Client‐owned dogs of any sex, breed, or reproductive status with a new histologic diagnosis of carcinoma were eligible for inclusion. Dogs were excluded from the study if they had received any anti‐inflammatory medications including nonsteroidal anti‐inflammatory drugs or glucocorticoids of any form (PO, topical, ophthalmic) within 30 days of diagnosis. Dogs also were excluded if they had received any cytotoxic or metronomic chemotherapeutic agents within 30 days of diagnosis. Dogs with acute, inflammatory comorbidities that commonly incite an acute phase inflammatory response were excluded from the study. These comorbidities included: acute pancreatitis, pneumonia, immune‐mediated diseases such as immune‐mediated hemolytic anemia or immune‐mediated thrombocytopenia, acute gastroenteritis, acute hemorrhagic diarrhea syndrome, or acute kidney injury. Additionally, dogs with a concurrent diagnosis of hyperadrenocorticism were excluded because of endogenous hypercortisolism. Last, dogs with multiple neoplastic diagnoses were excluded.

Control dogs were recruited from the community, and health status was determined based on collection of a medical history and performance of a complete physical examination. Control dogs were required to be healthy and free of any active underlying disease process. Control dogs were excluded if they were receiving any medications other than monthly preventives. Complete blood counts were performed in all control dogs, which were excluded if anemia, leukocytosis (or any increase in individual leukocyte types) were identified. Control dogs were excluded if they were of any breed with increased risk of diagnosis of carcinoma, including breeds associated with urothelial carcinoma (Scottish Terrier, West Highland White Terrier, Wire‐haired Fox Terrier, Beagle, and Shetland Sheepdog), breeds associated with platelet size and number abnormalities (Cavalier King Charles Spaniels and sighthounds), or dogs of primarily white hair coats because of their risk of squamous cell carcinoma. Client consent was obtained for this study and the protocol was approved by the institutional animal care and use committee (Protocol #1901001843).

### 2.2 Study design

All dogs diagnosed with carcinoma had blood collected as part of their diagnostic evaluation with excess serum used for the evaluation of inflammatory and thrombopoietic markers. Healthy control dogs had blood collected after physical examination. All blood samples were collected by peripheral venipuncture into 2 tubes: a lavender top tube anticoagulated with EDTA and a serum tube. Lavender top tubes were used for a CBC and blood smear analysis and performed by medical technologists using a commercial analyzer (Advia 2021I, Siemens Medical Solutions Inc, Malvern, Pennsylvania). Serum was separated by centrifugation at 3000*g* for 10 minutes and transferred to a cryovial for storage in an −80°C freezer.

Based on CBC results, dogs were stratified for subgroup analysis based on the presence or absence of thrombocytosis. Thrombocytosis was defined as a platelet count ≥500 × 10^3^/μL. Serum IL‐6 concentrations were measured using a commercial ELISA (Quantikine ELISA Canine IL‐6 Immunoassay, R&D Systems Inc, Minneapolis, Minnesota). Interkeukin‐6 concentrations were measured using a quantitative sandwich enzyme immunoassay technique based on color change. The intensity of color change then was compared to a standard curve using a microplate reader (Synergy HT multi‐detection microplate reader, Biotek U.S., Winooski, Vermont). The detection range for canine IL‐6 is 6.1 to 2000 pg/mL.^30^ The stability of serum IL‐6 at −80°C has been shown to be 6 months.^31^

Serum TPO concentrations were measured using a commercial ELISA kit (Canine TPO ELISA kit, mybiosource.com, San Diego, California), or more specifically by quantitative double‐antibody sandwich technique resulting in colorimetric change and comparison to a standard curve. The detection range for canine TPO is 15.6 to 1000 pg/mL. Serum TPO stability at −80°C is 3 months.^32^ Serum samples were batched and run at 3‐month intervals so as to preserve stability of IL‐6 and TPO.

### 2.3 Statistical analysis

A statistical power analysis was performed for sample size estimation, based on prior evaluation of IL‐6 and TPO concentrations in people with carcinoma‐associated thrombocytosis.^5^ In this study, IL‐6 and TPO were present at significantly increased mean concentrations (33.42 ± 74.3 pg/mL and 34.43 ± 119.1 pg/mL, respectively) in people with ovarian carcinoma and thrombocytosis when compared to people with normal platelet counts (13.71 ± 25.61 pg/mL and 16.2 ± 42.61 pg/mL, respectively).^5^ This calculation yielded a need for ≥55 individuals to identify a difference in IL‐6 and TPO concentrations between groups when set at 80% power.

Data were analyzed using commercial software (Stata, StataCorp LLC, College Station, Texas). Normality was assessed using the Shapiro‐Wilk test. Because numerical distributions in most comparison groups violated the assumption of normality, summary measures are reported as median [range]. Results of selected numeric variables (platelet count, IL‐6 concentration, TPO concentration) were compared between carcinoma and control groups, and between carcinoma with thrombocytosis and carcinoma with normal platelets groups, using Wilcoxon rank sum tests for pairwise comparisons. Correlation was assessed by calculating Spearman rank correlation coefficients. A value of *P* < .05 was considered significant.

## 3 RESULTS

One hundred sixteen dogs were included in the study: 63 were diagnosed with histologically confirmed carcinoma and 53 dogs were healthy controls. Originally, 55 healthy dogs were included, but in analysis it was identified that 2 were Beagle mixed breed dogs, and these dogs subsequently were excluded. The dogs with carcinoma were significantly older than those in the control group (*P* < .01; Table 1). No significant difference was found in sex distribution between groups. Several breeds were represented in both groups, with the most common in both groups being mixed breed dogs (Table 1). Thirty‐nine of 63 dogs with carcinoma (61.9%) had no reported comorbid conditions. The comorbid conditions reported in the other 24 dogs with carcinoma (38.1%) included lower urinary tract infections (6/24), chronic kidney disease (CKD, 5/24), myxomatous valvular disease (4/24), diabetes mellitus (DM, 3/24), and hypothyroidism (2/24). Other comorbid conditions noted in individual dogs included chronic hepatitis, keratoconjunctivitis sicca, chronic rhinitis, and chronic enteropathy.

**TABLE 1:** Demographic information and median [range] platelet count for the dogs with carcinoma, subclassified based on the presence or absence of thrombocytosis (defined as a platelet count ≥500 × 10^3^/μL), as well as demographic information and median [range] platelet count of the group of healthy control dogs

	Age (years)	Sex	Breeds (listed if ≥3)	Platelet count (× 10^3^/μL)
Carcinoma (N = 63)	10 [2‐15]	30 FS 1 FI 30 M 2 MI	Mixed Breed (18), Beagle (5), Labrador Retriever (5), Shih Tzu (3), West Highland White Terrier (3)	349 [161‐660]
With normal platelets (N = 51)	11 [7‐18]	23 FS 1 FI 25 MN 2 MI	Mixed Breed (15), Beagle (4), Labrador Retriever (5)	310 [161‐464]
With thrombocytosis (N = 12)	8.5 [0.33‐14]	7 FS 5 MN	Mixed Breed (3), Shih Tzu (3)	548.5 [533‐660]
Control (N = 53)	7 [1‐14]	19 FS 7 FI 23 MN 4 MI	Mixed Breed (15), Australian Shepherd (4), Golden Retriever (4), Labrador Retriever (4), Pembroke Welsh Corgi (3)	266 [146‐459]

Abbreviations: FI, female intact; FS, female spayed; MI, male intact; MN, male neutered.

Platelet counts for dogs with carcinoma (median, 349 × 10^3^/μL; range, 161‐660) were significantly higher than the platelet counts for the healthy control dogs (median, 266 × 10^3^/μL; range, 146‐459; *P* < .001). Twelve of the 63 dogs (19.6%) with carcinoma had thrombocytosis. None of the healthy dogs had thrombocytosis. The median [range] platelet count of dogs with carcinoma and thrombocytosis was 548.5 × 10^3^/μL [533‐660]. The mean platelet volume (MPV) was significantly higher in the carcinoma group (median, 9.8; range, 7.5‐16.8) than the control group (median, 9.2; range, 7.2‐11.9; *P* = .002). Median MPV did not differ between dogs with carcinoma and thrombocytosis (median, 9.45; range, 7.6‐12.1) and dogs with carcinoma and normal platelet counts (median, 9.5; range, 7.2‐16.8; *P* = .54). No other differences were identified on hematology or biochemical profiles when comparing groups. Eight of the dogs with carcinoma and thrombocytosis had documented comorbid conditions including CKD (n = 3), and 1 each with chronic hepatitis, keratoconjunctivitis sicca, myxomatous valvular disease, chronic rhinitis, and atopic dermatitis.

**FIGURE 1:**
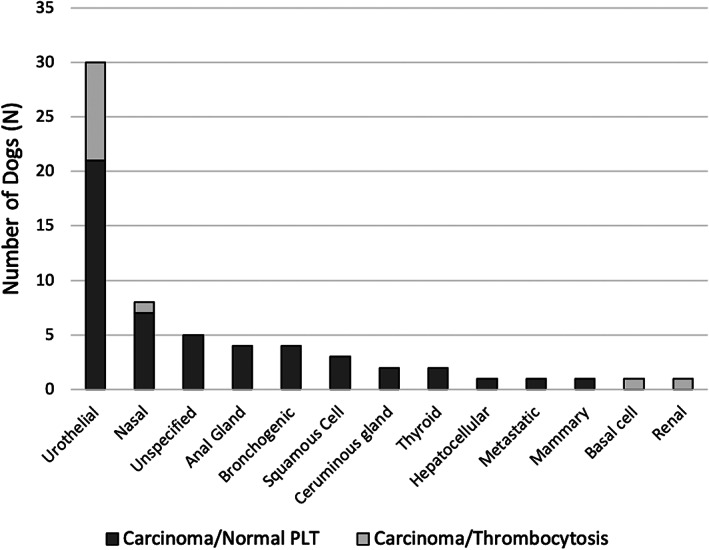
Bar graph depicting the histologic diagnoses of carcinoma in dogs. The entire bar represents the total number of dogs diagnosed with that carcinoma type. The dark bars represent the dogs with carcinoma and a normal platelet count. The light bars represent the dogs with carcinoma and thrombocytosis

The most common carcinoma diagnosed was urothelial carcinoma (30/61, 49.2%), with nasal carcinoma, apocrine gland anal sac adenocarcinoma, and bronchogenic carcinoma being the next most frequently diagnosed (each 4/61, 6.6%). Of the 12 dogs with carcinoma and thrombocytosis, 9 were diagnosed with urothelial carcinoma, with the other 3 being nasal carcinoma, renal carcinoma, and basal cell carcinoma, respectively (Figure 1).

**FIGURE 2:**
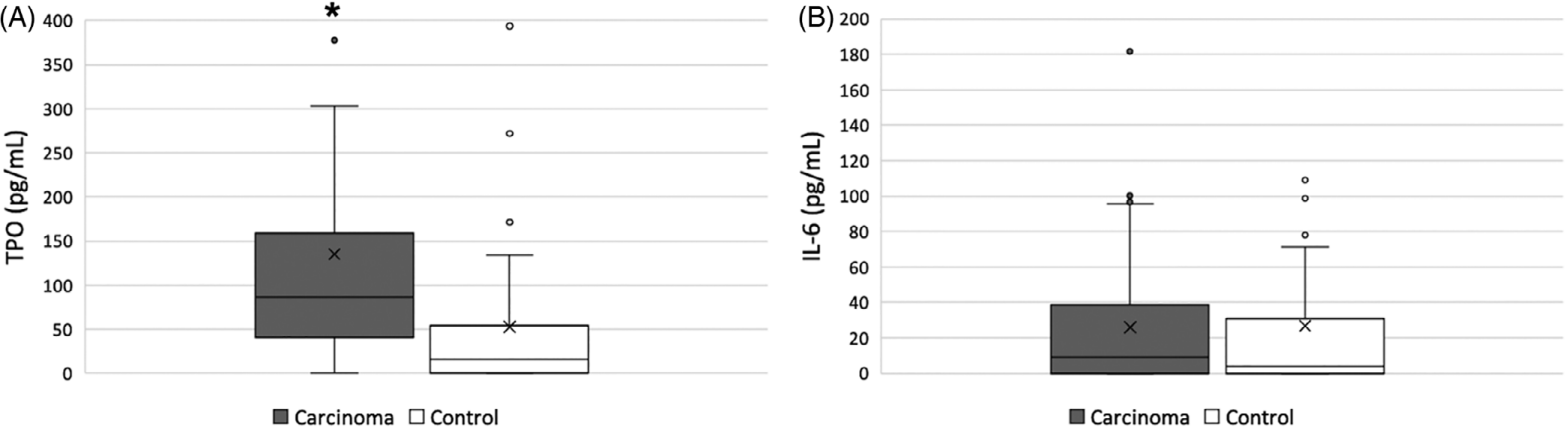
Box and whisker plot depicting the concentrations of TPO (A) and IL‐6 (B) in dogs with carcinoma and healthy control dogs. The box represents the first and third quartiles with the middle line representing the median. The whiskers represent the range out to 1.5x the interquartile range (IQR), with outliers depicted by individual data points. The concentration of TPO (median [range]) was significantly higher in dogs with carcinoma when compared to healthy dogs (*P* < .001). The concentration of IL‐6 (median [range]) was not different between dogs with carcinoma compared to controls (*P* = .16). Significant differences are depicted with the “*” symbol. IL‐6, interleukin‐6; TPO, thrombopoietin

**FIGURE 3:**
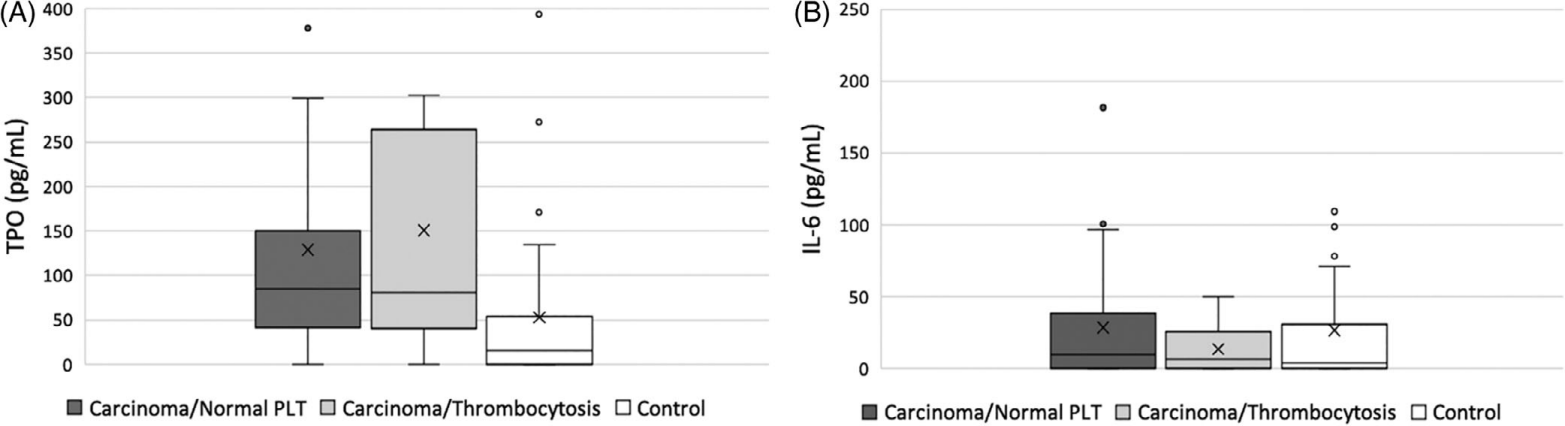
Box and whisker plot depicting the concentrations of TPO (A) and IL‐6 (B) in dogs with carcinoma and a normal platelet count (Carcinoma/Normal platelet [PLT]), dogs with carcinoma and thrombocytosis (Carcinoma/Thrombocytosis) and healthy control dogs. The box represents the first and third quartiles with the middle line representing the median. The whiskers represent the range out to 1.5x the IQR, with outliers depicted by individual data points. The concentrations of TPO and IL‐6 (median [range]) were not different when comparing dogs with carcinoma/normal PLT to dogs with carcinoma/thrombocytosis (*P* = .11 and *P* = .54, respectively). IL‐6, interleukin‐6; TPO, thrombopoietin

Median [range] TPO concentrations were significantly higher in the dogs with carcinoma (87.4 pg/mL [0 to >600]) when compared to controls (15.8 pg/mL [0 to >600]; *P* < .001; Figure 2). Median [range] TPO concentrations were not different in dogs with carcinoma when comparing those with thrombocytosis (80.2 pg/mL [0 to >600]) to those with a normal platelet count (86.2 pg/mL [0 to >600]; *P* = .11; Figure 3). Median [range] IL‐6 concentrations were not different between dogs with carcinoma (9.7 pg/mL [0‐181.5]) and control dogs (3.9 pg/mL [0‐280.8]; *P* = .16; Figure 2). Median [range] IL‐6 concentrations were not different in dogs with carcinoma when comparing those with thrombocytosis (6.3 pg/mL [0‐50.0]) to those with a normal platelet count (6.0 pg/mL [0‐280.8]; *P* = .54; Figure 3). No correlation was found between TPO or IL‐6 concentrations and platelet count, hematocrit, leukocyte count, or age.

## 4 DISCUSSION

Serum TPO concentrations and platelet count were increased in dogs with carcinoma compared to healthy dogs. This finding suggests carcinoma‐associated thrombopoiesis, but thrombocytosis was noted infrequently. In our study, thrombocytosis was noted in 20% of dogs with carcinoma. This frequency is similar to what has been observed in people, in which 10% to 50% of patients with carcinoma have concurrent thrombocytosis.^5^, ^12^, ^13^, ^14^, ^15^, ^16^

Thrombopoietin is a cytokine that is mainly generated in the liver and works in an endocrine manner to stimulate megakaryocytes in the bone marrow, spleen, and other extramedullary sites to increase numbers of platelets.^22^, ^23^, ^24^, ^25^, ^26^, ^27^, ^28^ In health, TPO concentration is mediated by platelet load.^22^, ^26^, ^27^, ^28^ Platelets have substantial capacity to absorb, metabolize, and destroy TPO, thus regulating the amount of TPO in circulation.^26^, ^27^, ^28^ Neoplastic manipulation of TPO is not completely understood, but it is hypothesized that carcinoma stimulates the production of excess TPO by the production of inflammatory mediators, specifically IL‐6.^5^, ^7^, ^10^, ^29^, ^33^ Interleukin‐6 was not increased in our population of dogs with carcinoma, and thus other inflammatory mediators likely affect platelet production and consumption in dogs with carcinoma.

Thrombopoietin was increased in dogs with carcinoma regardless of platelet count, and the majority of dogs with carcinoma had platelet counts within the reference interval. It is possible that the tumor microenvironment or other paraneoplastic and inflammatory conditions led to substantial platelet consumption, and thus TPO production contributed to maintenance of a normal platelet count. The dogs with carcinoma did not have evidence of inflammation that would constitute an acute phase response, but comorbid conditions were noted in 38.1% of dogs with carcinoma that could have impacted the balance of platelet production and consumption. Additionally, although inflammatory conditions have been commonly indicated in thrombocytosis, non‐neoplastic and noninflammatory diseases recently have been described in association with thrombocytosis in dogs, including CKD, valvular disease, and DM.^3^, ^4^ These conditions were present in low numbers in our study, and their impact on TPO concentrations is unknown. Future studies should exclude all comorbid conditions (inflammatory and noninflammatory), but this can be difficult to control in a largely geriatric population of dogs.

Interleukin‐6 is produced by carcinomas in people, and is hypothesized to stimulate excess production of TPO.^5^, ^7^ In our study, IL‐6 concentrations were not increased in dogs with carcinoma, and no difference was found in IL‐6 concentration when dogs with carcinoma were stratified by platelet count. In dogs, IL‐6 is reliably increased in models of sepsis and systemic inflammatory response syndrome, but IL‐6 concentrations tend to normalize within 6 days of an inciting inflammatory event, whereas other acute inflammatory markers such as C‐reactive protein and tumor necrosis factor α (TNF‐α) remained increased for 14 to 21 days after the inciting event.^34^ Therefore, it is possible that in our population of dogs with carcinoma, collection of blood at a single time point did not allow for complete evaluation of the influence of IL‐6. Numerous cytokines have been shown to affect thrombopoiesis such as platelet alpha granule, platelet‐derived growth factor, transforming growth factor β (TGF‐β), interleukin‐1 (IL‐1), interleukin‐3 (IL‐3), interleukin‐4 (IL‐4), interleukin‐11 (IL‐11), TNF‐α, granulocyte‐macrophage colony stimulating factor, and fibroblast growth factor 2.^29^, ^33^, ^34^ Further investigation of inflammatory cytokines in dogs with carcinoma may help elucidate the factors that drive increased platelet production.

Mean platelet volume was significantly increased in the carcinoma group when compared to the control group. Mean platelet volume is a commonly used morphological parameter that is determined using a machine‐calculated platelet volume.^35^, ^36^, ^37^, ^38^ In health, platelet volume has been shown to be inversely proportional to platelet count.^39^ Mean platelet volume has been shown to be a significant indicator of inflammation in various types of cancer in people, both independent of and when compared to platelet count.^35^, ^36^ However, in inflammatory conditions, MPV can be discordant with platelet count, as was the case in our patient population.^37^, ^38^ In people, measurement of immature platelet fraction can help differentiate among causes of increased destruction or consumption of platelets and decreased production of platelets.^35^, ^36^, ^37^, ^38^ This approach may be a more direct method for evaluation of factors affecting platelet count in dogs that could be evaluated in future studies.

Our study identified an increase in TPO in dogs with carcinoma, but an association with thrombocytosis was not observed. This disparity is likely a consequence of small sample size and overrepresentation of certain tumors in our study. Our study had an overrepresentation of urothelial carcinoma because of institutional research programs. Power for the study was calculated based on expected differences extrapolated from people with ovarian carcinoma. Although it is a urogenital tumor, ovarian cancer is uncommonly diagnosed in dogs, and tumor behavior is likely different when comparing different carcinomas in dogs. Future studies should expand upon the increase in TPO identified in dogs with urothelial carcinoma to narrow the scope to this single tumor type. Additionally, comorbid diseases in these dogs likely impacted platelet count, both positively and negatively, but future studies will be necessary to understand if these diseases affect TPO concentration.

Given that thrombocytosis creates a favorable environment for neoplastic cells, it represents an intriguing and important abnormality to investigate. Thrombopoietin is increased in dogs with carcinoma regardless of platelet count, but is likely only 1 component of a more complex pathophysiology in the tumor environment of carcinoma in dogs.

## CONFLICT OF INTEREST DECLARATION

George E. Moore serves as Consulting Editor for Experimental Design and Statistics for the *Journal of Veterinary Internal Medicine*. He was not involved in review of this manuscript. No other authors have a conflict of interest.

## OFF‐LABEL ANTIMICROBIAL DECLARATION

Authors declare no off‐label use of antimicrobials.

## INSTITUTIONAL ANIMAL CARE AND USE COMMITTEE (IACUC) OR OTHER APPROVAL DECLARATION

Authors declare no IACUC or other approval was needed.

## HUMAN ETHICS APPROVAL DECLARATION

Authors declare human ethics approval was not needed for this study.
